# Supplementary figures and images for: A computational strategy for finding novel targets and therapeutic compounds for opioid dependence
Source: PLoS One. 2018 Nov 7;13(11):e0207027. doi: 10.1371/journal.pone.0207027 (PMC6221321; doi:10.1371/journal.pone.0207027)

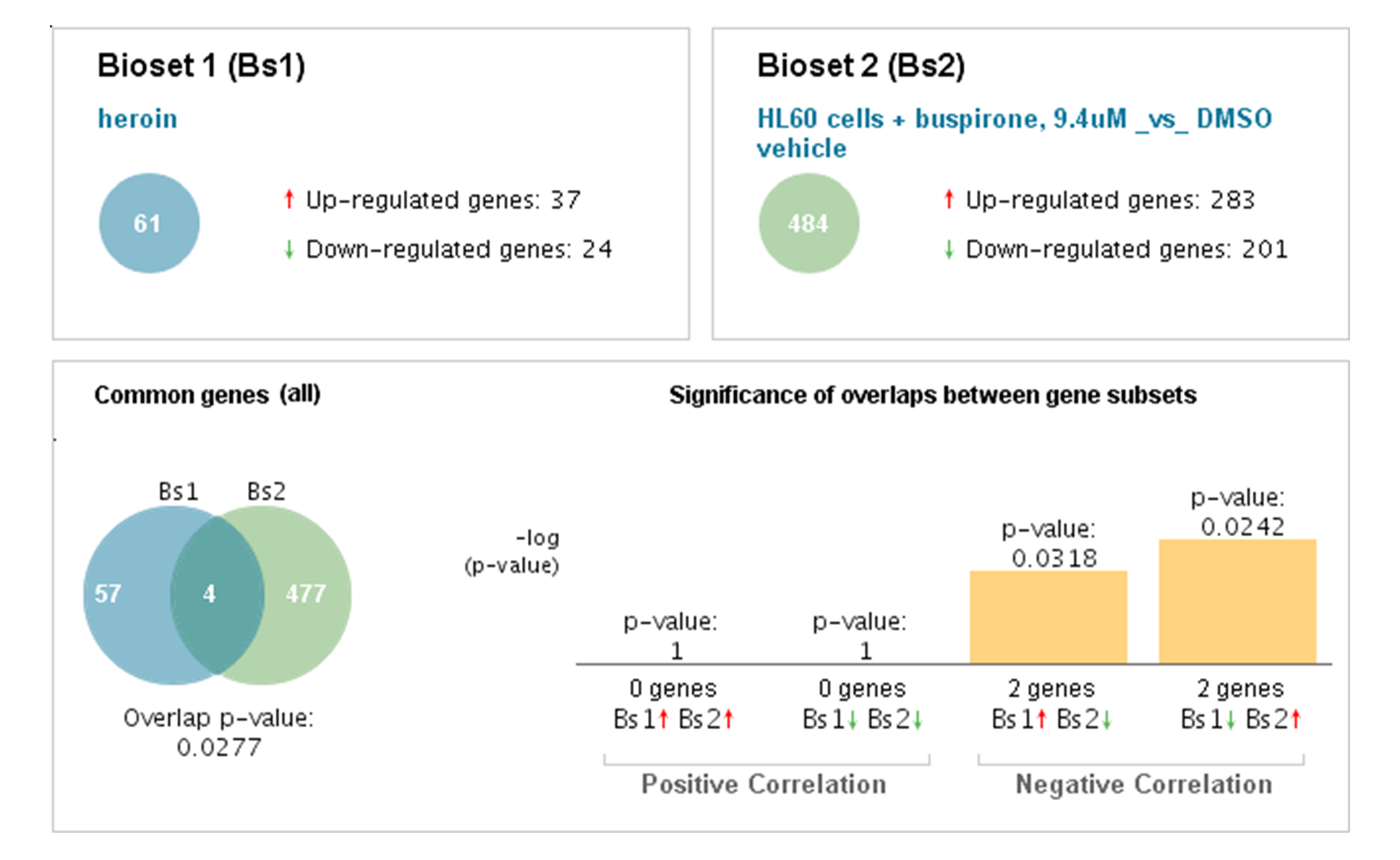

Supplement: S1 Fig — Four genes were regulated by both heroin and buspirone, but in opposite directions. (TIF) [file pone.0207027.s001.tif]
