# Supplementary material for: A computational strategy for finding novel targets and therapeutic compounds for opioid dependence
Source: PLoS One. 2018 Nov 7;13(11):e0207027. doi: 10.1371/journal.pone.0207027 (PMC6221321; doi:10.1371/journal.pone.0207027)
Supplement: S6 Table — (DOCX) [file pone.0207027.s007.docx]

**S6 Table: Significantly enriched transcription regulators and associated harmful effects after morphine exposure.** Each associated harmful effect was given an association score (AS) defined as the sum of the maximum fold changes of the associated DEGs that the corresponding transcription regulator regulated. The DEGs regulated by each factor are also shown in this table. Note that all of the abbreviations used in this table can be found in the legend of Table 1.

| Transcription Regulators | Type | Known Regulators (Literature Support) | Associated Harmful Effects (AS) | No. of Regulated DEGs | Regulated DEGs | Phase |
| --- | --- | --- | --- | --- | --- | --- |
| CTCF (HM) | Epigenetic Reg | Y [1] | Chronic (6.99) | 5 | Cntd1, Nedd9, Rasl11a, Serad4, Rasd1 | Up-IE |
|  |  |  | Dep (2.97) | 2 | Klf2, Kcnk13 |  |
|  |  |  | Phys harm (2.78) | 2 | Nedd9, Rasl11a |  |
| SIN3A | Epigenetic Reg | Y [2] | Chronic (6.38) | 5 | Cntd1, Nedd9, Rasl11a, Serad4, Rasd2 | Up-IE |
|  |  |  | Dep (2.9) | 2 | Klf2, Kcnk19 |  |
|  |  |  | Pleasure (3.77) | 3 | Cntd1, Kcnk13, Scrn3 |  |
| EZH2 (HM) | Epigenetic Reg |  | Dep (2.9) | 2 | Klf2, Kcnk14 | Up-IE |
| SUZ12 | Epigenetic Reg |  | Dep (1.2) | 1 | Kcnk20 | Up-IE |
| E2F1 | TF |  | Dep (2.9) | 2 | Klf2, Kcnk15 | Up-IE |
|  |  |  | Pleasure (3.77) | 3 | Cntd1, Kcnk13, Scrn3 |  |
| POLR2A (HM) | RNA Polymerase II | Y [3] | Dep (2.9) | 2 | Klf2, Kcnk16 | Up-IE |
|  |  |  | Phys harm (2.78) | 2 | Nedd9, Rasl11a |  |
|  |  |  | Pleasure (3.77) | 3 | Cntd1, Kcnk13, Scrn3 |  |
| TAF1 | TATA box binding protein | Y [4] | Dep (2.9) | 2 | Klf2, Kcnk20 | Up-IE |
|  |  |  | Pleasure (3.77) | 3 | Cntd1, Kcnk13, Scrn3 |  |
| MEF2C | TF | Y [3] | Dep (1.7) | 1 | Klf2 | Up-IE |
| SIN3A | Epigenetic Reg | Y [2] | Dep (6.46) | 4 | Acer2, Baiap2, Lhx3, Nsun7 | Up-M |
| MEF2A | TF | Y [3] | Phys dep (5.34) | 4 | Btrc, Elac1, Nup133, Tamm41 | Up-M |
| BRF2 | TF |  | Phys dep (1.39) | 1 | Tamm41 | Up-M |
| POL2 | RNA Polymerase II |  | Dep (3.89) | 3 | Col4a6, Numb, Scara3 | Up-L |
| HDAC6 | Epigenetic Reg | Y [3] | Psycho dep (1.21) | 1 | Cyp26a1 | Down-IE |
| E2F6 (HM) | TF |  | Phys dep (17.93) | 13 | Acsl4, Ankrd34a, Anp32b, Glcci1, Grin1, Hist1h3b, Nup50, PPP6c, Purg, Rbm12, Sdf2l1, Sox18, Zscan22 | Down-M |
| SAP30 | Epigenetic Reg | Y [5] | Phys dep (15.49) | 11 | Ankrd34a, Anp32b, Glcci1, Grin1, Hist1h3b, Ppp6c, Purg, Rbm12, Sdf2l1, Sox18, Zscan22 | Down-M |
| ZKSCAN1 | TF |  | Phys dep (6.81) | 5 | Anp32b, Hist1h3b, Ppp6c, Rbm12, Zscan22 | Down-M |
| NR4A1 | NR4a1 | Y [6] | Phys dep (1.57) | 1 | Sdf2l1 | Down-M |
| ZBTB33 | Epigenetic Reg |  | Phys dep (11.41) | 8 | Anp32b, Hist1h3b, Nup50, Ppp6c, Purg, Sdf2l1, Sox18, Zscan22 | Down-M |
| HDAC8 | Epigenetic Reg | Y [3] | Acute (1.78) | 1 | Cldn5 | Down-M |
|  |  |  | Phys dep (6.67) | 4 | Cldn5, Rbm12, Sdf2l1, Sox18 |  |
| HDAC6 | Epigenetic Reg | Y [3] | Acute (1.78) | 1 | Cldn5 | Down-M |
|  |  |  | Phys dep (4.84) | 3 | Cldn5, Hist1h3b, Sdf2l1 |  |

1. Higgins GA, Allyn-Feuer A, Athey BD. Epigenomic mapping and effect sizes of noncoding variants associated with psychotropic drug response. Pharmacogenomics. 2015;16(14):1565-83. Epub 2015/09/05. doi: 10.2217/pgs.15.105. PubMed PMID: 26340055.

2. Godino A, Jayanthi S, Cadet JL. Epigenetic landscape of amphetamine and methamphetamine addiction in rodents. Epigenetics. 2015;10(7):574-80. Epub 2015/05/30. doi: 10.1080/15592294.2015.1055441. PubMed PMID: 26023847; PubMed Central PMCID: PMCPMC4622560.

3. Robison AJ, Nestler EJ. Transcriptional and epigenetic mechanisms of addiction. Nat Rev Neurosci. 2011;12(11):623-37. Epub 2011/10/13. doi: 10.1038/nrn3111. PubMed PMID: 21989194; PubMed Central PMCID: PMCPMC3272277.

4. dela Pena I, Kim HJ, Sohn A, Kim BN, Han DH, Ryu JH, et al. Prefrontal cortical and striatal transcriptional responses to the reinforcing effect of repeated methylphenidate treatment in the spontaneously hypertensive rat, animal model of attention-deficit/hyperactivity disorder (ADHD). Behav Brain Funct. 2014;10:17. Epub 2014/06/03. doi: 10.1186/1744-9081-10-17. PubMed PMID: 24884696; PubMed Central PMCID: PMCPMC4077266.

5. Tabuchi A. Synaptic plasticity-regulated gene expression: a key event in the long-lasting changes of neuronal function. Biol Pharm Bull. 2008;31(3):327-35. Epub 2008/03/04. PubMed PMID: 18310887.

6. Campos-Melo D, Galleguillos D, Sanchez N, Gysling K, Andres ME. Nur transcription factors in stress and addiction. Front Mol Neurosci. 2013;6:44. Epub 2013/12/19. doi: 10.3389/fnmol.2013.00044. PubMed PMID: 24348325; PubMed Central PMCID: PMCPMC3844937.
