# Supplementary material for: A computational strategy for finding novel targets and therapeutic compounds for opioid dependence
Source: PLoS One. 2018 Nov 7;13(11):e0207027. doi: 10.1371/journal.pone.0207027 (PMC6221321; doi:10.1371/journal.pone.0207027)
Supplement: S7 Table — (DOCX) [file pone.0207027.s008.docx]

**S7 Table: Significantly enriched transcription regulators and associated harmful effects after heroin exposure.** Each associated harmful effect was given an association score (AS) defined as the sum of the maximum fold changes of the associated DEGs that the corresponding transcription regulator regulated. The genes regulated by each factor are also shown in this table. Note that all of the abbreviations used in this table can be found in the legend of Table 1.

| Transcription Regulators | Type | Known Regulators (Literature Support) | Associated Harmful Effects (AS) | No. of Regulated DEGs | Regulated DEGs | Phase |
| --- | --- | --- | --- | --- | --- | --- |
| POLR2A (HM) | RNA Polymerase II | Y [1] | Acute (7.56) | 5 | Csrnp1, Stab1, Rasl11a, Dapk1, Ap5m3 | Up-IE |
|  |  |  | Phys harm (17.56) | 12 | Ace, Actr2, Elac2, Fdps, Hif1an, Khsrp, Kif23, Klf2, Man2b2.Ppp6r1, Rasl11a, Rpl7l1, Tbc1d2b |  |
|  |  |  | Psycho dep (6.52) | 4 | Baiap2, Dapk1, Plekhf1, Ptbp1 |  |
|  |  |  | Soc harm (24.74) | 18 | Actr2, Cep350, Dnajb12, Fdps, Hif1an, Rasl11a, Khsrp, Kif23, Lemd2, Man2b2, Mok, Ncs1, Plekhg3, Ppp6r1, Rpl7l1, Tbc1d2b, Tfap2a, Trappc1 |  |
| EGR1 | TF | Y [1] | Psycho dep (6.52) | 4 | Baiap2, Dapk1, Plekhf1, Ptbp1 | Up-IE |
|  |  |  | Soc harm (24.4) | 18 | Actr2, Cep350, Dnajb12, Extl1, Fdps, Khsrp, Kif23, Lemd2, Man2b2, Mok, Ncs1, Plekhg3, Ppp6r1, Rpl7l1, Sez6, Tbc1d2b, Tfap2a, Trappc1 |  |
| CREB1 | TF | Y [1] | Phys harm (16.04) | 11 | Actr2, Elac2, Fdps, Hif1an, Khsrp, Kif23, Klf2, Man2b2.Ppp6r1, Rpl7l1, Sez6, Tbc1d2b | Up-IE |
|  |  |  | Psycho dep (6.52) | 4 | Baiap2, Dapk1, Plekhf1, Ptbp1 |  |
|  |  |  | Soc harm (20.45) | 15 | Actr2, Cep350, Dnajb12, Fdps, Hif1an, Khsrp, Kif23, Lemd2, Man2b2, Mok, Ppp6r1, Rpl7l1, Sez6, Tbc1d2b, Trappc1 |  |
| MAX | TF |  | Acute (7.56) | 5 | Csrnp1, Stab1, Rasl11a, Dapk1, Ap5m2 | Up-IE |
|  |  |  | Psycho dep (6.52) | 4 | Baiap2, Dapk1, Plekhf1, Ptbp1 | Up-IE |
| CCNT2 | TF |  | Psycho dep (6.52) | 4 | Baiap2, Dapk1, Plekhf1, Ptbp1 | Up-IE |
| E2F6 (HM) | TF |  | Psycho dep (6.52) | 4 | Baiap2, Dapk1, Plekhf1, Ptbp1 | Up-IE |
| MAZ | TF |  | Psycho dep (6.52) | 4 | Baiap2, Dapk1, Plekhf1, Ptbp1 | Up-IE |
| CTCF (HM) | Epigenetic Reg | Y [2] | Acute (7.56) | 5 | Csrnp1, Stab1, Rasl11a, Dapk1, Ap5m1 | Up-IE |
|  |  |  | Psycho dep (6.52) | 4 | Baiap2, Dapk1, Plekhf1, Ptbp1 |  |
| CHD2 | Epigenetic Reg |  | Psycho dep (6.52) | 4 | Baiap2, Dapk1, Plekhf1, Ptbp1 | Up-IE |
| PHF8 | Epigenetic Reg |  | Psycho dep (6.52) | 4 | Baiap2, Dapk1, Plekhf1, Ptbp1 | Up-IE |
| EZH2 (HM) | Epigenetic Reg |  | Psycho dep (2.5) | 2 | Fbrsl1, Tfap2d | Down-IE |
| SUZ12 | Epigenetic Reg |  | Psycho dep (2.5) | 2 | Fbrsl1, Tfap2d | Down-IE |

1. Robison AJ, Nestler EJ. Transcriptional and epigenetic mechanisms of addiction. Nat Rev Neurosci. 2011;12(11):623-37. Epub 2011/10/13. doi: 10.1038/nrn3111. PubMed PMID: 21989194; PubMed Central PMCID: PMCPMC3272277.

2. Higgins GA, Allyn-Feuer A, Athey BD. Epigenomic mapping and effect sizes of noncoding variants associated with psychotropic drug response. Pharmacogenomics. 2015;16(14):1565-83. Epub 2015/09/05. doi: 10.2217/pgs.15.105. PubMed PMID: 26340055.
