# Supplementary material for: A computational strategy for finding novel targets and therapeutic compounds for opioid dependence
Source: PLoS One. 2018 Nov 7;13(11):e0207027. doi: 10.1371/journal.pone.0207027 (PMC6221321; doi:10.1371/journal.pone.0207027)
Supplement: S8 Table — (DOCX) [file pone.0207027.s009.docx]

**S8 Table: Supporting evidence of involvement of transcription regulators in drug dependence and addiction from literature.** Our results on dependence-associated regulatory factors, GO terms, KEGG pathways, and identified small compounds can be found in S6, S7, S9, S10 Tables. Note that all of the abbreviations used in this table can be found in the legend of Table 1.

|  | Evidence from literature | Our results (transcription regulators) | Our results  (GO, KEGG) | Our results  (identified small compounds) |
| --- | --- | --- | --- | --- |
| Morphine | GluR1, cAMP-PKA pathway [1]  cAMP-CREB pathways [2] |  | Regulation of alpha-amino-3-hydroxy-5-methyl-4-isoxazole propionate selective glutamate receptor activity (GO term, down-M) |  |
|  | MEF2 [2] | MEF2C (up-IE, dep) MEF2A (up-M, phys dep) |  | Benserazide (MEF2A), Securinine (MEF2C), Isocorydine (MEF2A),  Gabapentin (MEF2A) |
|  | NFKB [2] |  | Negative regulation of NF-κB transcription factor activity (GO term, up-L) |  |
|  | Epigenetics | HDAC6 (down-IE, psycho dep); HDAC6/HDAC8 (down-M, phys dep) |  | Gabapentin (HDAC6) |
|  | p38 MAPK pathway; CREB pathway [3] ERK signaling/pathway [4] |  | MAPK signaling pathway (KEGG, down-M) |  |
| Heroin | Direct and indirect striatal pathways (Fos gene) [5] Pathway initiated by CCL3/4/5 binding to CCR5 (c-Fos) [3] ΔFOSB [2] | C-FOS (up-M, pleasure) FOSL2 (down-M, pleasure) |  |  |
|  | cAMP-PKA pathway [1] CREB [2] | CREB1 (up-IE, psycho dep) | Response to cAMP (GO term, down-M) | Pirlindole (CREB1), Amoxapine (CREB1), Scopolamine (CREB1), Meclofenoxate (CREB1) |
|  | NFKB [2] |  | Positive regulation of I-κB kinase/NF-κB signaling (GO term, down-L) |  |
|  | EGRs [2] | EGR1 (up-IE, Psycho dep) |  | Pirlindole (EGR1), Amoxapine (EGR1), Meclofenoxate (EGR1) |
|  | STATs [2] | STAT1 (up-L, pleasure) STAT1/STAT2 (down-IE, pleasure) |  |  |
|  | Epigenetics |  | Regulation of histone modification (GO term, up-M) |  |
|  | MAPK/ERK pathway |  | Regulation of MAP kinase activity (GO term, down-L) |  |
|  | c-Jun involved in morphine [3] FosB + c-Jun induced by drugs of abuse [2] | C-JUN (up-M, pleasure) | Regulation of JNK cascade (GO term, down-L) |  |

1. Ron D, Jurd R. The "ups and downs" of signaling cascades in addiction. Sci STKE. 2005;2005(309):re14. Epub 2005/11/10. doi: 10.1126/stke.3092005re14. PubMed PMID: 16278489.

2. Robison AJ, Nestler EJ. Transcriptional and epigenetic mechanisms of addiction. Nat Rev Neurosci. 2011;12(11):623-37. Epub 2011/10/13. doi: 10.1038/nrn3111. PubMed PMID: 21989194; PubMed Central PMCID: PMCPMC3272277.

3. Jacobsen JH, Hutchinson MR, Mustafa S. Drug addiction: targeting dynamic neuroimmune receptor interactions as a potential therapeutic strategy. Curr Opin Pharmacol. 2016;26:131-7. Epub 2015/12/15. doi: 10.1016/j.coph.2015.10.010. PubMed PMID: 26657076.

4. Melik Parsadaniantz S, Rivat C, Rostene W, Reaux-Le Goazigo A. Opioid and chemokine receptor crosstalk: a promising target for pain therapy? Nat Rev Neurosci. 2015;16(2):69-78. Epub 2015/01/16. doi: 10.1038/nrn3858. PubMed PMID: 25588373.

5. Badiani A, Belin D, Epstein D, Calu D, Shaham Y. Opiate versus psychostimulant addiction: the differences do matter. Nat Rev Neurosci. 2011;12(11):685-700. Epub 2011/10/06. doi: 10.1038/nrn3104. PubMed PMID: 21971065; PubMed Central PMCID: PMCPMC3721140.
